# The fragmentation of responsible AI: sector variation in organisational AI policies and statements of principle

**DOI:** 10.1007/s00146-026-03175-7

**Published:** 2026-06-27

**Authors:** Hannah Guy, Andrew Cox, Nitika Bhalla, Kate Miltner, Denis Newman-Griffis

**Affiliations:** 1 School of Information, Journalism and Communication, University of Sheffield, Sheffield, United Kingdom; 2 Centre for Machine Intelligence, University of Sheffield, Sheffield, United Kingdom

**Keywords:** Responsible AI, Ethical AI, AI policy, United Kingdom

## Abstract

The AI landscape has been changing dramatically with the growth of generative AI, and many governments, sector bodies, and individual organisations are working in parallel to stake out their own visions of ethical and responsible AI use. Statements, guidelines, and policies on responsible AI have proliferated in the last five years, yet clarity remains elusive on what ‘responsible AI’ means or how to put it into practice. Research to date has not examined how the principles and practices of responsible AI may vary across sector, an increasingly important concern as AI use grows throughout the economy and society. In this article, we empirically examine inter- and intra-sectoral variance in the principles articulated for responsible AI in organisational policies. We analysed 80 documents from organisations in 8 sectors, focussing on policies current at the time of the 2024 AI Seoul Summit. Our content analysis identified 31 distinct principles in these policies, only ten of which appeared in more than 50% of the documents. We found clear sectoral differences in the principles invoked for responsible AI, as well as the audiences who were intended to engage with putting those principles into practice. Our analysis focussed on organisations shaping responsible AI in a single nation, the United Kingdom, but our findings illustrate the admixture of national and international actors affecting AI practice. Our findings show that responsible AI is increasingly fragmented, and that an understanding of sector-level variation is essential to shaping the future of responsible AI.

**Supplementary Information:**

The online version contains supplementary material available at https://doi.org/10.1007/s00146-026-03175-7.

## Introduction

Adoption of artificial intelligence (AI) technologies has accelerated dramatically across many sectors since the paradigm shift moment of ChatGPT’s public release in November of 2022, making powerful generative AI technologies publicly available and easily accessible at a scale not seen before. However, AI use is deeply controversial, and a wide range of concerns have been expressed about the ethical, social, political, and environmental implications of wider AI adoption in society (Jobin et al. 2019; Corrêa et al. 2023). Many governmental organisations, professional sector bodies, and individual organisations and experts have attempted to intervene by publishing statements defining approaches to developing, using, and/or governing AI. These documents use varying language of ethical AI, responsible AI, trustworthy AI, and more (Tollon and Vallor 2025). They are issued in various forms, as guidelines (European Commission *Ethics guidelines for trustworthy AI*), statements of principle (OECD *AI principles overview*), legislation (EU *AI Act*), government statements (Seoul Declaration), and public positions (UN *Seizing the opportunities of safe, secure and trustworthy artificial intelligence systems for sustainable development*). However, translating these documents into behavioural change in practice remains a significant challenge, and this proliferation creates a cacophony of voices putting forth many competing visions of what AI use should look like.

In this study, we anatomise this cacophony to better understand the evolving shape of responsible AI (RAI) at a moment of dramatic transformation. We analyse published statements to identify commonalities and differences in how actors seek to define and shape RAI in their own contexts, and to guide the actions of their own diverse groups of stakeholders. In contrast to previous studies, which have sought to characterise the shape of RAI as a whole (Corrêa et al 2023; Hagendorff 2024), we investigate differences in how RAI is represented and understood between different sectors, reflecting the increasing fragmentation of AI use for diverse purposes across a wide range of contexts, from education to media to health.

We rigorously analysed 80 statements issued by organisations seeking to shape AI practice across a variety of sectors, focussing on a moment of historic change leading up to the Seoul AI Safety Summit in May 2024. We take the UK, which positioned itself as an international AI leader during this period by establishing the first AI Safety Summit, as the primary context for our study by focussing on organisations seeking to shape AI policy and practice in UK professional sectors. However, our findings reflect the dual nature of responsible AI discourse as both global (with key documents from the UN, European Commission, and G7, as well as multinational corporations) and local (e.g. individual UK universities, legal organisations, and others), with clear relevance for international debates around RAI. Our results reveal a fragmentation of RAI definitions and understandings across organisations and sectors, contrasting with prior claims of converging understandings of responsible AI (Fjeld et al. 2020). Our study illustrates the vital need for organisations and sectors to approach RAI as dynamically constructed and constantly evolving, as opposed to a single, static set of guiding principles, and to build responsible practices that are based in their specific AI uses and the contexts in which they operate.

The language of AI policies and research is varied and highly contested (Tollon and Vallor 2025), but we adopt the language of ‘responsible AI’ to reflect our focus on the overall understanding, behaviours, and practices which shape AI use, including ethical considerations, trustworthiness, and interpersonal and societal impact. Responsible AI is multiple in nature and contextual in practice as it is shaped by many different decisions informing the use of AI in specific applications (Newman-Griffis 2025). Our study aimed to characterise this multiplicity through the lens of organisational statements around AI, focussing on three key research questions:

1. What are the most common principles informing responsible AI across the analysed sectors and organisations, and which are relatively neglected?
2. What are the similarities and differences concerning the ways in which different sectors approach responsible AI?
3. How have advances in publicly available generative AI systems affected perceptions of responsible AI?

By examining these questions, our study provides a new understanding of the fragmentation and variability of responsible AI across organisational and sectoral contexts. Our analysis illustrates the ways in which individual organisations focus on distinct dimensions of responsible AI, and distinct groups of actors who are able to shape responsible AI in practice. Section 2 establishes the context of our study, illustrating the gap in sector-based understanding in previous literature examining documentary evidence of approaches to responsible AI. Section 3 presents our content analysis methodology and our emphasis on the language used directly by organisations rather than imposing our own structured interpretation. Our key findings, including novel evidence of variations in document audience, RAI principles, and sectoral patterns, are presented in Sect. 4, and placed in the wider context of current challenges in operationalising responsible AI in Sect. 5. We conclude by reflecting on the simultaneous challenges this variation presents for wider responsible AI action and the value of fragmented understandings in context-sensitive responsible AI practice.

## Literature review

As adoption of AI and awareness of its risks have grown, there have been many attempts to define RAI. ‘Responsible AI’ has a complex history reflecting competing efforts between industry actors, social researchers, policy experts, and others to craft actionable language and demonstrable behaviours alongside the discourses of AI ethics and trustworthiness (Tollon and Vallor 2025). Our study aims to understand how these diverse and competing understandings of responsible AI are materialised in guiding documents published by individual organisations. We therefore summarise here the key studies that have laid the groundwork for our analysis of responsible AI statements and policies, and illustrate that this growing body of research still has a significant gap in understanding how and why these documents may vary between organisational and sector contexts. Our study provides the missing empirical evidence to fill this gap, and demonstrates why responsible AI must be viewed also through a lens of multiple and fragmentary practice.

Academic research has proposed guidelines and principles, government policies and regulations have set initial shapes for expectations and accountability structures around AI use (e.g. EU AI Act), sector lead bodies have put forward collective statements (e.g. Russell Group of Universities, News Media Association), and many individual organisations have published their own AI policies to present their position on the contentious issue of AI use (a number of which are addressed in our data collection, see Sect. 3.1 below). However, previous research has shown that practitioners attempting to define, implement, govern, or otherwise achieve RAI face a key barrier in the operationalisation of ethical principles in practice, due to their abstract nature (Madaio et al. 2020; Mokander and Floridi 2023; Morley et al. 2019; Ryan et al. 2022) and the lack of consensus over definitions of ethical principles (Pant et al. 2024). Principles are also seen as open to multiple interpretations and lack conceptual and institutional unity (Metcalf and Moss 2019). The nature of specific AI applications has also been suggested as a key differentiator for ethical considerations, with some practitioners advocating for sector- and application-specific definitions of what is ethical, arguing that generalised statements have limited use (Ibanez and Olmeda 2022).

Responding to this need for shared, actionable understanding, a number of previous studies have analysed the many published statements of principles that seek to define what constitutes RAI (see Table 3 below for a summary of some of the main studies). In a widely cited, seminal paper Jobin et al. (2019) analysed 84 AI policy documents published before April 2019. They found a wide range of principles being proposed. However, the most frequently occurring were “Transparency”, followed by “Justice, fairness and equity”, “Non-maleficence”, “Responsibility and accountability” and “Privacy”. Each of these principles was used by the authors to refer to a range of terms that they had grouped together. Because more than half of all documents mention these five principles, the authors suggested a convergence around these, with six less common principles falling outside this consensus. However, the “Justice, fairness and equity” category in particular includes many other specific codes (*Justice, fairness, consistency, inclusion, equality, equity, (non-) bias, (non-) discrimination, diversity, plurality, accessibility, reversibility, remedy, redress, challenge, access and distribution*). Each of these terms is also open to multiple interpretations: for example, bias is often presented as a specific problem of data and AI with a purely technical meaning, but also refers to wider socio-political biases. “Fairness” and “justice” are general terms for what is good and have been debated for centuries. The authors also acknowledge differences in contextual meaning ascribed to the principles which would diminish the sense of consensus.

Fjeld et al. (2020) analysed 36 prominent sets of AI principles published between September 2016 and October 2019. Eight themes were identified: “Fairness and non-discrimination, privacy, accountability, transparency and explainability, safety and security, professional responsibility, promotion of human values and human control of technology”. It is evident that there are common terms with Jobin et al. (2019). Furthermore, the authors argue that “the conversation around principled AI is beginning to converge, at least amongst the communities responsible for the development of these documents” (4). However, we suggest that the intent of the paper is precisely to synthesise and combine commonly used terms, rather than to observe and describe the range in use, so the sense of an emerging consensus is questionable. Also, the authors themselves emphasise that “principles should be understood in their cultural, linguistic, geographical, and organisational context,” (5) pointing to variation in meaning beneath the surface.

In the same year as Fjeld et al. (2020) were writing, Hagendorff (2020) published an examination of 22 AI guidelines from 2015 to 2020. The most frequent “key issues” were, jointly, “privacy protection” and “fairness, non-discrimination, justice”, followed by “accountability”, “transparency, openness”, and “safety, cybersecurity” (102). Notably, again we see fairness, transparency and privacy occurring towards the top principles/themes across documents. However, Hagendorff also lists 16 other principles beyond the common five. Interestingly, despite the smaller sample in use than in other studies, the sense of the range of terms is greater. Hagendorff also reflects on the many societal concerns that are rarely mentioned in any of the documents, such as computer sentience, impact on social cohesion, political abuse of AI, lack of diversity in the AI community, and hidden social and ecological costs. This points to the need to interrogate what is not mentioned in such sets of principles as much as what is.

Another large-scale study was undertaken by Corrêa et al. (2023). They present a detailed analysis of 200 AI ethics guidelines from across the globe, mostly published before the end of 2022. They identify 17 different principles across the corpus. They found “Transparency/Explainability/Auditability” to be the most frequently mentioned term, followed by five others that occur in more than 50% of the documents: “Reliability/Safety/Security/Trustworthiness” and “Justice/Equity/Fairness/Non-discrimination”, “Privacy”, “Accountability/liability”, and “Freedom/Autonomy/Democratic values/Technological sovereignty” (13). Again, there are many familiar terms in this list. However, as the category labels suggest, the terms were collected together, in this case on the basis of ngram analysis. The researchers suggest that transparency is becoming “the dominant topic of concern” (16), whereas previous research in 2014 and 2016 they believed found Fairness and Accountability to be the most-cited principles. However, this is to ignore the seeming proliferation of terms that appear to be important for at least some documents.

Following up on his 2020 study, Hagendorff (2024) undertook a further examination of the ethics of AI, with the purpose of seeking to identify whether generative AI had shifted the agenda. He aimed to cover “a significant proportion of the existing literature on the ethics of generative AI” (2); thus, he included a much wider scope of the types of documents and papers collected than only organisational statements of principle. In total, he collected 179 documents from 2021 or after, using 29 key terms related to generative AI and, through content analysis, identified “19 distinct clusters of ethics topics” (4). “Fairness—Bias” was found to be the most discussed issue, followed by “Safety”, “Harmful content—Toxicity”, “Hallucinations”, and “Privacy” (5). The issue of transparency was still detected in the dataset, but only in a minority of documents. These findings suggest that, whilst ethics related to generative AI largely reflect ethics related to AI in general, AI policies/guidelines that specifically address generative AI may prioritise other issues/concepts over others. For example, harm may not be considered as pressing in the context of generative AI compared to AI overall.

This body of research illustrates the challenge of consistently defining RAI, and of navigating how it changes in different contexts and over time. The studies discussed here have used different data collection methods, time frames, and approaches to analysis, limiting their direct comparability. In addition, most documents analysed have not been formally published, making them difficult to identify systematically through bibliographic searches or to develop a comprehensive list of statements. Nonetheless, it is evident that some principles recur quite commonly, such as transparency, fairness, and privacy. There does appear to be a core of recurrent concerns. Yet this masks that often large numbers of terms have been grouped together in studies under an author-chosen heading. The long tail of less common terms also reveals a lack of consensus. In addition, several of the authors hint that the terms are used to mean different things in different contexts. Furthermore, the major shift of technology capability with generative AI appears also to have changed how the issues are seen. In this context, a sense of emerging consensus dissolves.

Our study follows in this vein with updated data and a focus on characterising and understanding the variation in the terminology used to define responsible AI. In undertaking further research in this topic, our study sought to explore the suggestion made by a number of authors that the sectors in which AI policies are produced, and the audiences they are produced for, may lead to important differences in how RAI is represented. Moreover, by analysing AI policies at the sector level, we aimed to enrich our understanding of the variability of RAI, and how it may be contingent in policy and practice on the different contexts in which it is implemented. We also sought to take the discussion forward with more recent data, particularly to explore evidence for the shift of attention caused by the current focus on generative AI.

As in many other countries, the dramatic advances and new investments in AI technology have prompted a wide range of hopes and fears in the UK society. Successive UK governments and many other organisations (e.g. the UK’s National Health Service) have issued strategy documents hoping to maximise the benefits of AI. Interest and concern have been greatly amplified by the arrival of generative AI. AI is widely predicted to have a significant impact on the UK economy and society, though the precise character and speed of change remain uncertain (Department for Education,^2024^). The impact is likely to be felt differently in different parts of the economy. For example, amongst the sectors most exposed from an employment perspective are information and communication and education, as well as finance and insurance, and public administration (Department for Education 2024). At the same time as offering many benefits, AI has raised widespread concerns. Some are technical, many are ethical (Anwar et al. 2024).

## Methodology

Our study used a content analysis approach to analyse 80 policy documents issued by 66 individual organisations, governments and intergovernmental organisations, and sector bodies (Sect. 3.1). We analysed the distinct principles and concepts discussed in these documents in relation to responsible use and impacts of AI technologies (Sect. 3.2).

### Data collection and sampling

The central aim of our study was to collect and analyse AI policy documents to examine the range of sectoral positions on RAI in the period immediately leading up to and since the introduction of commercially available generative AI as a moment of dramatic transformation in adoption and practice of AI use worldwide. We follow similar methodological approaches to previous studies reviewed in the previous section in examining the frequency of occurrence of ethical principles and terms. However, in building our collection, we differ from prior research in three key ways:

*Sector-level analysis* We purposely sampled documents setting out AI principles and policies from eight professional sectors (Table 1) with significant influence in the development, application, communication, and governance of responsible AI. We used inclusive sector groupings (e.g. Governmental and Inter-governmental Organisations, Health) to enable us to analyse documents issued by organisations and bodies serving similar roles with regard to responsible AI rather than separating based on the nature or structure of organisations.

**Table 1:** Sectors analysed in this study, with the number of documents included from each and the number of unique organisations authoring those documents

Sector	Reason for inclusion	Example organisations	No. of documents (organisations)
Governmental and Inter-governmental Organisations	As central actors in promoting national AI strategy and with a potential concern for the wider societal benefits and impacts	UK government, European Commission, United Nations	16 (10)
Health	AI has been identified as having potentially powerful applications in the health sector, but there are also important concerns about how sensitive data will be handled	NHS, Bupa, World Health Organisation	4 (4)
Higher Education	Generative AI has particularly significant implications for university learning, research and administration	JISC, Russell Group, individual universities	15 (15)
Legal	AI has significant legal ramifications in itself, e.g. for copyright, and in addition is being widely adopted by law firms	Ashurst, Clifford Chance, Linklaters	6 (6)
Media & Information	AI is increasingly being used in the media and also the media has a strong role in discussing the controversies around AI	BBC, Elsevier, News Media Association	7 (6)
Multinationals (non-tech)	With AI’s ability to analyse large datasets, consultancy firms and professional services are increasingly applying AI to their work. They also offer guidance on RAI to other organisations	KPMG, Deloitte, Accenture, Rolls Royce	10 (8)
Research & Professional Organisations	Certain technology-based research and professional organisations seek to advise on AI use	The Alan Turing Institute, Ada Lovelace Institute, The Royal Society	6 (6)
Technology	Companies driving the development of AI	Google, Microsoft, Meta, Intel	16 (11)
Total			80 (66)

*Reference timepoint* We sampled documents that were current at the time of the Seoul AI Safety Summit in May 2024 as a representation of current thinking and approaches two years into wide-scale adoption of commercial generative AI.

*Geographical context* To facilitate our sectoral analysis, we focussed specifically on the UK, which positioned itself through leadership of the AI Safety Summit series as a key global actor for responsible AI. Our sample reflects the dual global and local nature of AI development and use, including UK-specific organisations (e.g. the BBC, UK universities and legal firms) as well as Intergovernmental Organisations shaping AI regulation and multinational corporations building AI technologies.

These three emphases provide essential grounding for our analysis in the particular contexts and ways in which AI technologies were being used at a particular point in time. This allows us to examine the context-sensitive nature of responsible AI in practice, in contrast with previous studies that have been agnostic to these differences.

Having chosen the sectors to study, we used the team’s local knowledge to identify key organisations for each sector based on a combination of market share/breadth of audience (e.g. preferring large organisations with national or international reach), reputation in shaping practice and culture within a sector (e.g. Big Four consultancy firms, the BBC as a media leader), and direct relevance to AI policy and practice in the UK (e.g. The Alan Turing Institute as the UK’s national institute for AI). We searched the websites of these organisations to locate their current policy statement(s) regarding AI. Some organisations had several such documents extant at the time of the search. Some had none that we could locate. For example, whilst many of the leading technology companies have produced at least some form of statement on RAI, and some had more than one, several leading media organisations had not published anything. The UK’s leading media outlets, such as the BBC, Channel 4, The Guardian, Sky News, Times, Guardian, etc., were examined; however, only the BBC had published their approach to AI. At the same time, some organisations indicated that they adhered to another organisation's approach to AI use. For example, across the legal sector, whilst some of the UK’s top law firms have published their AI policies, others referred to the Competition & Markets Authority’s publication on AI, indicating that they endorsed the approach set out there. In our search of websites for UK higher education institutions, we found that many universities had no publicly available policy. These factors, combined with sector size and the varying role of AI in each sector (and of each sector in AI), explain why some sectors are represented by more organisations than others in our sample. Table 1 offers a summary of the sectors represented in the collection, and Appendix 1 provides a full list of the documents used.

The collected documents represent a snapshot of the position in the mid-2024. The sample sought to update the picture from previous research discussed in the literature review and also to represent the potential re-evaluation of AI since the launch of generative AI tools such as ChatGPT at the end of November 2022. The particular cut-off point we chose was the AI Seoul Summit on the 21 st and 22nd of May 2024. The Summit, led by the G7, brought together international governments, AI companies, and academics to advance global discussions on AI. This event marked the latest stage in international governmental considerations of AI safety risks and AI opportunities. Whilst the aim in our study was to capture the most up-to-date perspectives on AI policy, some of these guidelines dated back to 2019. These policies/guidelines appeared to be the most recent documents produced by organisations concerning AI, and so, whilst they were older than our aimed timeframe, it was deemed more important to include whatever perspectives these organisations had, rather than omit their approach to AI entirely.

### Method of analysis

Content analysis was used to analyse the sampled documents. As the literature review above has shown, previous studies that have sought to examine organisations’ AI guidelines have used a similar method to investigate the topic. This analytical approach is considered to be a rigorous, reliable, and replicable method used to count trends, patterns, and differences (Krippendorff 2018) and “the presence and frequency of specific terms, narratives or concepts” in data (Seale & Tonkiss 2018, p. 404). This method involves the coding of data to produce comparable categories (Teddlie and Tashakkori 2009). Content analysis can be used to count the frequency of various things within data, ranging from the basic enumeration of the presence of certain words/phrases to a deeper interpretation of what the data mean and its categorisation into author given categories.

The documents were coded for:

- Organisation sector—the overarching sector the organisation falls into.
- Publication date—The year and month (if identifiable) in which the document was published. The Internet Archive’s Wayback Machine (https://web.archive.org/) was used on documents where the publication date was not present. This provided an approximate authoring date by identifying when the PDF or website appeared online.
- Country/region of the organisation—Where the organisation is headquartered, or the region the organisation operates in.
- Target audience—the organisation’s stated intended audience for their guidelines. In some cases, this was not stated, and so their target audience was tagged as ‘not specified’.
- Principles proposed—these were considered to be “all those values/goals/commitments/rights defined/supported by the institutions/authors that listed them in their guidelines” (Corrêa et al. 2023, p. 6). Notably, in addition to common concepts allied with responsibility, such as transparency and accountability, we observed several key dimensions reflecting the wider contexts in which AI technologies are used and their potential impacts, such as ‘human rights’ and ‘workforce displacement.’ Our analysis includes both underlying principles and dimensions of possible impact as key elements of how organisations present and understand responsible AI. The full list of principles is presented in Table 2.

**Table 2:** Principles of responsible AI analysed

Transparency	Bias/discrimination	Misuse	Lawfulness
Explainability	Inclusion/diversity	Human centeredness	Risk
Interpretability	Accessibility	Human rights	Privacy
Honesty	Empowerment	Harm	Security
Accountability	Well being	Sustainability	Safety
Responsibility	Accuracy	Workforce displacement	Robustness/rigour
Trust	Effective	Data Protection/agency	Stakeholders
Fairness	Reliability	Governance	

Different organisations presented their approach to RAI and the associated principles in differing structures and visual formats. Some documents presented principles as a list, whilst others published their approach in a more narrative form. Our method of categorising principles was consequently flexible to these differences in structure. With documents that listed principles, these were simple to identify and consequently to tag. With documents in paragraph form, when reading through the document, the coders pulled out the key principles or concepts that the organisation’s AI approach was organised around. Our approach sought to minimise the level of interpretation, but some choice and grouping by the coders was necessary.

We developed our set of principles inductively, drawing initially from sets of principles identified in the previous studies addressed in the literature review. To develop the set of principles, we followed an iterative process commonly used for inductive content analysis. The two researchers undertaking the analysis began reading through the documents and adding proposed principles to columns, noting that this principle was present in the document. After a small number of documents had been analysed, the two coders then consulted with each other to discuss their findings and the principles identified, determining which would be considered principles going forward and which would not, if two or more principles could be collapsed into one. This process continued as more documents were read and tagged with chosen terms becoming solidified when it became apparent that they were present across multiple documents. In total, 31 principles were identified and subsequently, all analysed documents were tagged for the occurrence of these principles. The principles identified are presented in Table 2.

Whilst it was found that many documents used the same term to describe a principle, for example, ‘Privacy’, ‘Security’, ‘Accountability’, etc., some principles were described using a variety of terms. In these cases, the coders conferred to determine whether these represented distinct principles or to settle on an agreed term. An example of this was the principle of ‘Sustainability’, which is often contested and described in a variety of ways: documents would refer to the idea of being environmentally sustainable either using the term sustainability or with different terms, such as ‘environmental protection’, ‘environmentally friendly’, ‘environmental impact’, etc. This was thus an interactive process, going back and forth between reading more documents, consultation, and then reworking the principle list if necessary. When the principles reached a saturation point where no further principles of note were identified, the remaining documents were read through and tagged for the set of principles.

We conducted a retrospective reliability test by a third coder recoding 13 randomly selected documents (including at least one from each of the eight categories) and comparing the results with the original coding. This achieved a 73% match. Given the number of items to code for (31) and the complexity of the documents we suggest this is a good score.

The principles, which include dimensions reflecting the wider contexts and potential impacts of AI use, were identified inductively during the document coding process, using previous studies as a starting point.

## Findings

Our collection consisted of a total of 80 items in total. The sector breakdown is presented in Table 1 above. In terms of the geographical locations of the examined organisations, the UK was the largest (45%, *n* = 36), followed by the USA (22.5%, *n* = 18), and global (19%, *n* = 15). There were also several smaller locations, such as the Netherlands (2.5%, *n* = 2), France (1%, *n* = 1), and Switzerland (1%, *n* = 1).

Section 4.1 presents our analysis of publication time, illustrating that whilst individual sectors have varied in their level of responsiveness and proactivity to AI, the launch of commercial generative AI has spurred on a major uptick of new documents across sectors. Section 4.2 illustrates the variation in audiences for responsible AI documents, highlighting the degree to which each sector tends to speak to itself and the wide variety of purposes for which responsible AI documents may be produced. Section 4.3 demonstrates the fragmentation of responsible AI principles across our collection, and Sect. 4.4 shows that different sectors vary dramatically in terms of what they represent as key aspects of responsible AI. Finally, Sect. 4.5 illustrates how the principles of responsible AI documents have demonstrably changed since the introduction of commercial generative AI, highlighting the evolving and contextual nature of responsible AI in practice.

### Publication date

The documents in our corpus were published at a fairly consistent rate, with a clear increase in publication frequency from ChatGPT’s launch in November 2022, Fig. 1 suggesting that many current policies specifically respond to the nature of generative AI. The earliest date of publication in our dataset is March 2019, with most documents (77.5%, *n* = 62) being published in 2023 or later and only 22.5% (*n* = 18) being published before 2023. The most common publication date was May 2024 (12.5%, *n* = 10); whilst only one of these documents was published specifically as part of the Seoul AI Safety Summit, it is possible that the timing of the Summit affected the timing of publication of other documents.

**Fig. 1:**
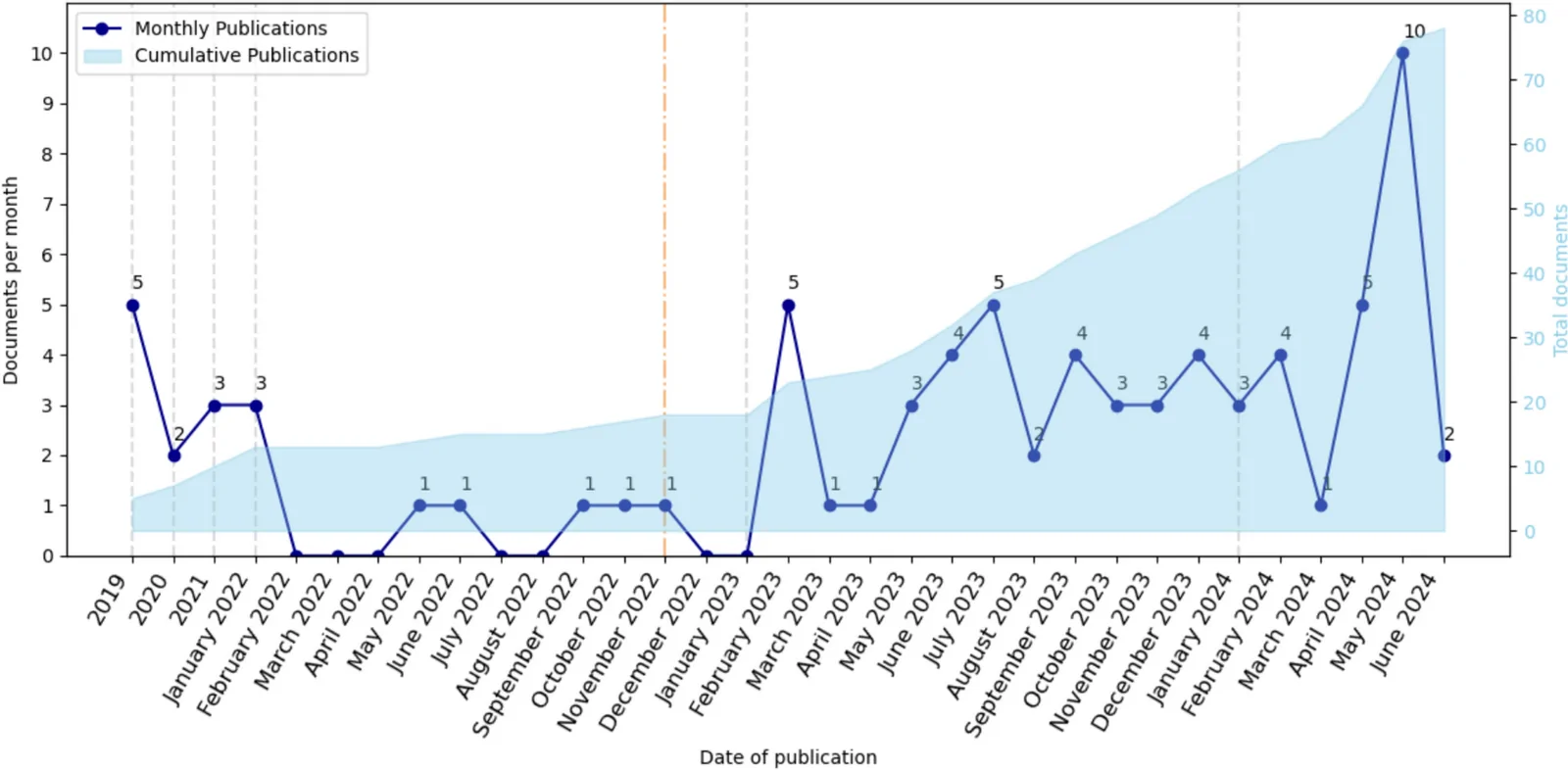
Number of documents in the analysed corpus by publication date. Documents published before 2022 are grouped by year, and from month by January 2022 onward. The cumulative number of documents published over time is shown alongside per-timepoint counts. Two documents were published in an undetermined month in 2024 and are excluded. Dashed lines indicate year boundaries, dot-dash line is the launch of ChatGPT in November 2022

**Fig. 2:**
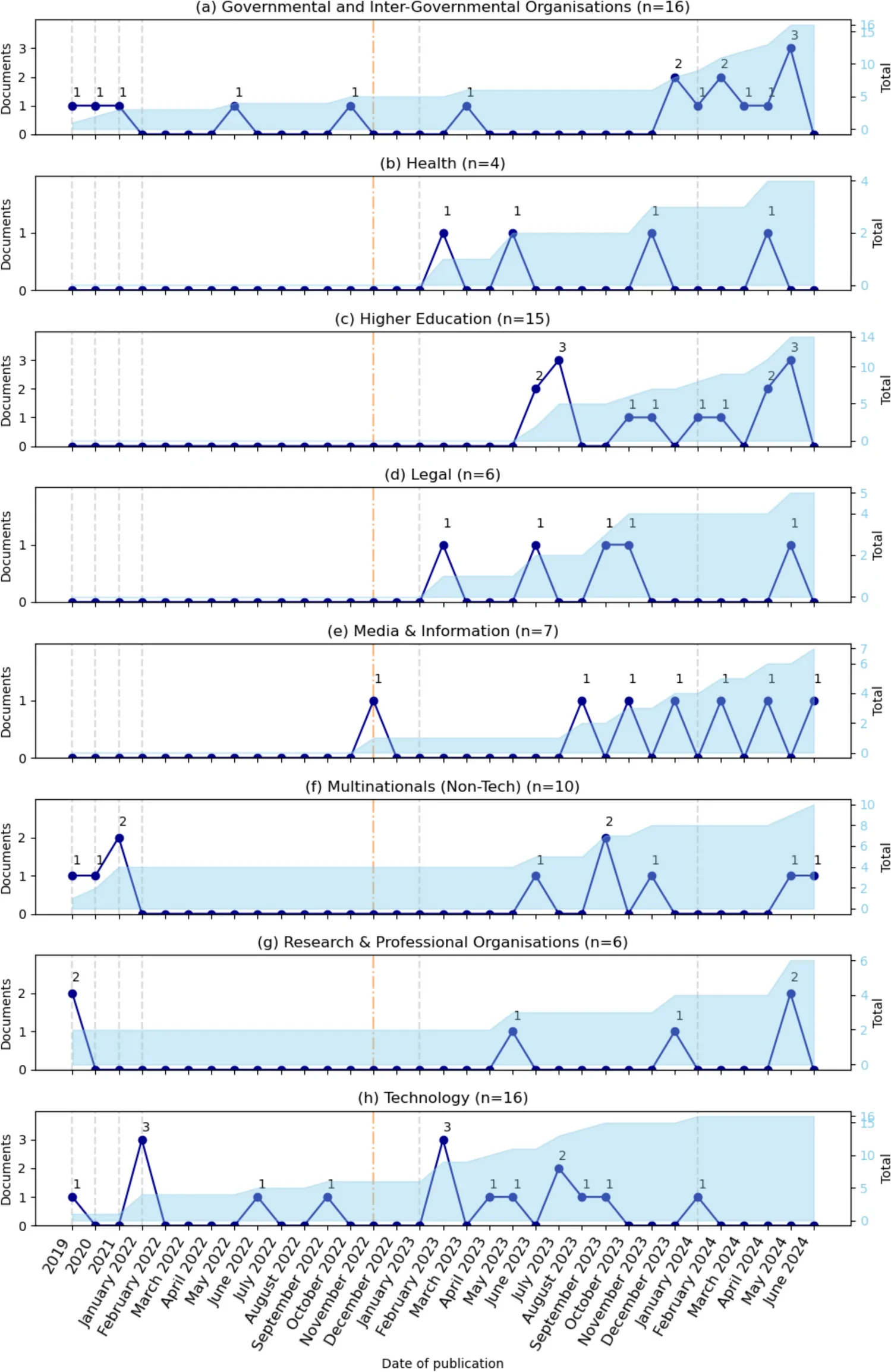
Number of documents in the corpus published per sector, by publication date. Documents published before 2022 are grouped by year, and from month by January 2022 onward. The cumulative number of documents published over time is shown alongside per-timepoint counts. Two documents were published in an undetermined month in 2024 and are excluded. Dashed lines indicate year boundaries, dot-dash line is the launch of ChatGPT in November 2022

At a sector level, however, we observed clear differences in when documents were published (Fig. 2). The documents for the Higher Education, Media & Information, Legal, and Health sectors were all published on or after the launch of ChatGPT in November 2022, reflecting the role of these documents as specific responses to the growth of generative AI. We did not observe clustering of documents being published in these sectors, aside from a small increase in HE policies during the summers between academic years. The spread of publication dates is more even in the technology sector (with 6 (37.5%) policy documents published before generative AI and 10 (62.5%) published after), publication dates still skew more post-genAI to pre-genAI; we observed a similar pattern for Governmental and Inter-governmental Organisations documents. It is important to note that these figures reflect publication of the *most current* policies available in May 2024, rather than *all* policies published during this period. Nonetheless, it is clear that the generative AI boom led to a period of active policy development in response, shaped by the needs and focus of individual sectors.

### Target audience

**Fig. 3:**
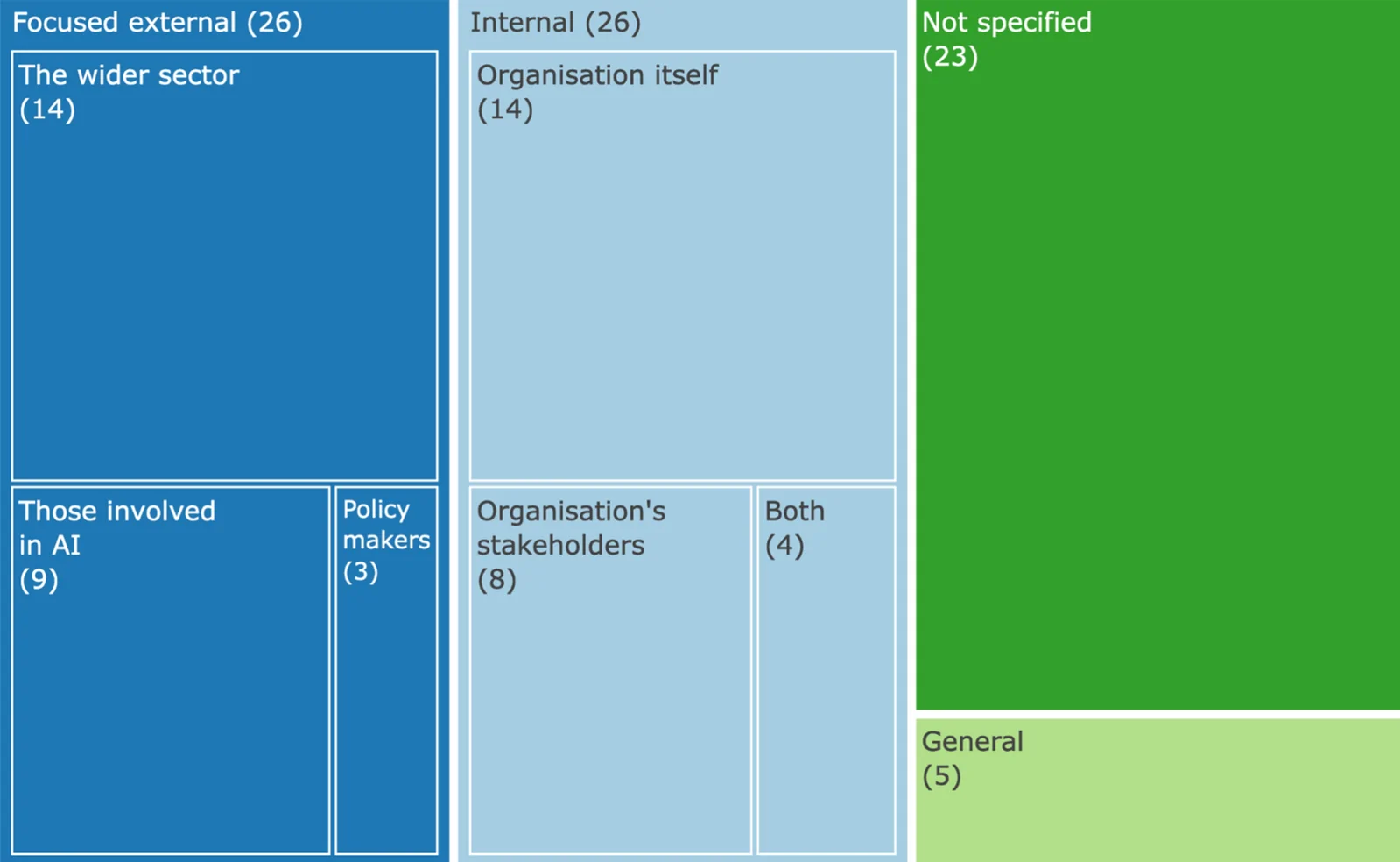
Target audiences for documents analysed, grouped by whether they are internal (i.e. directly related to the publishing organisation), a focussed external group, or other

Different responsible AI documents address different stakeholder groups, meaning that each provides a different snapshot of who is deemed relevant in responsible AI in practice. To better understand the scope of potential responsible AI stakeholders each individual document was intended to address, we also categorised documents based on their stated audience (Fig. 3). Over a quarter (29%, *n* = 23) did not specify their intended audience; this included general-purpose documents (e.g. broad statements from Intergovernmental Organisations, such as UNESCO and OECD) and documents where the audience is contextually implied, such as users of specific technologies (e.g. Microsoft guidance on Bing). This was followed jointly by the target audience being the organisation itself and the wider sector the organisation was a part of (17.5%, n = 14). 11% (*n* = 9) of documents were intended for those involved in AI, such as researchers, developers, and engineers. 10% (*n* = 8) of organisations targeted their stakeholders, and 5% (*n* = 4) targeted the organisation itself and its stakeholders. Finally, only 3 (4%) of the documents’ intended audience was policy-makers.

**Fig. 4:**
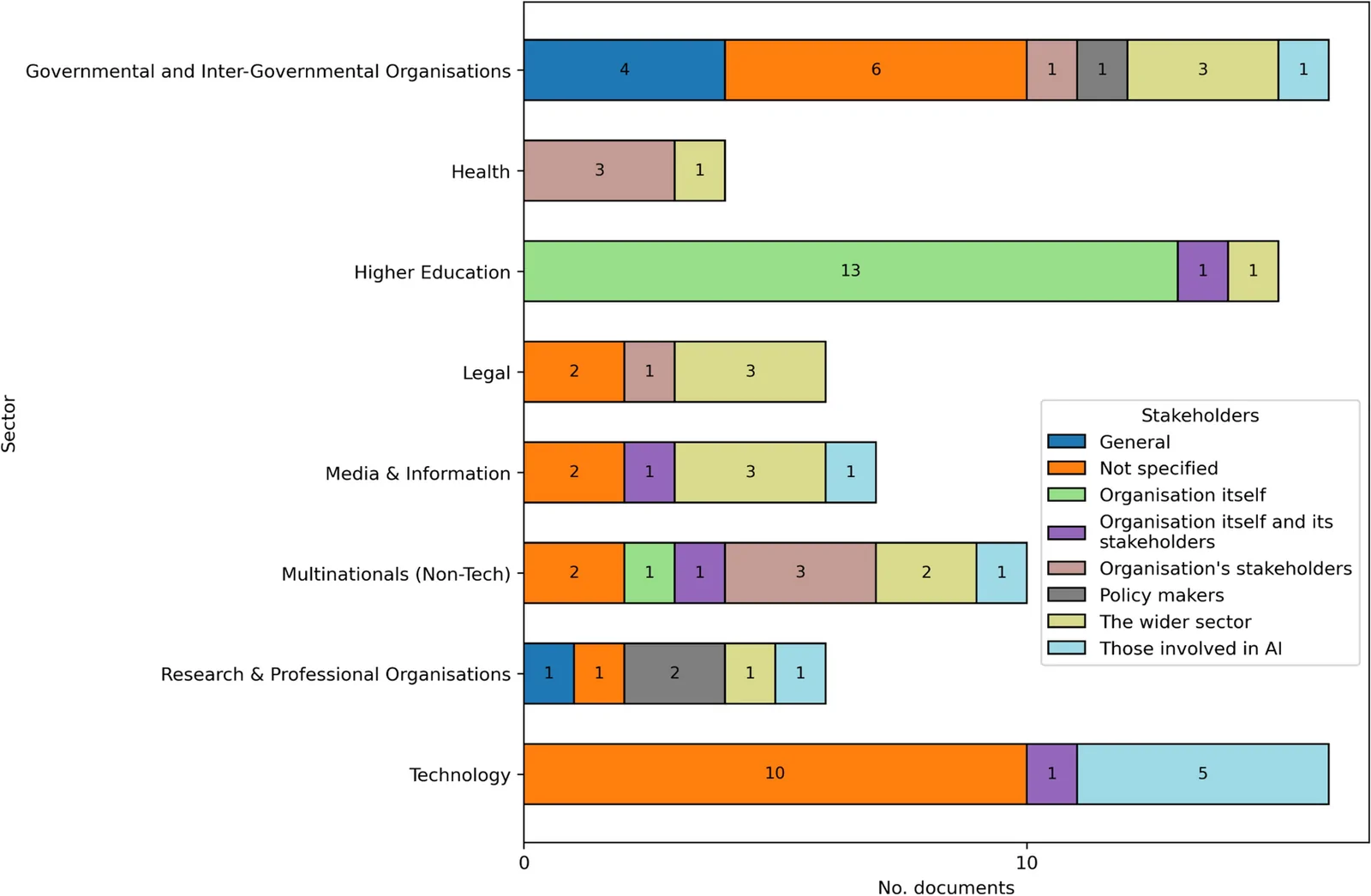
Target audience of analysed documents, by sector

We also considered how documents’ stated audiences varied by organisational sector (Fig. 4). It is interesting to note that the AI policy documents produced by Higher Education largely targeted just the organisation itself (*n* = 13, 86.5%). This explicit internal focus does not appear in the other sectors very often though it is possible some organisations had internally oriented policies on their intranets that we were not able to source. Another trend that stands out is that policy documents that target those involved in AI (e.g. developers, researchers, engineers etc.) were mostly produced by the technology sector, with 5 of the 9 (55.5%) doing so. Only Research & Professional Organisations and Governmental and Inter-governmental Organisations appeared to target policymakers, only accounting for 3 of the 80 (4%) policy documents. Overall, we observe that the clear majority of documents in most sectors address relatively narrow internal or sector-specific audiences, a factor possibly contributing to the proliferation of definitions of RAI.

### Most common principles identified

**Fig. 5:**
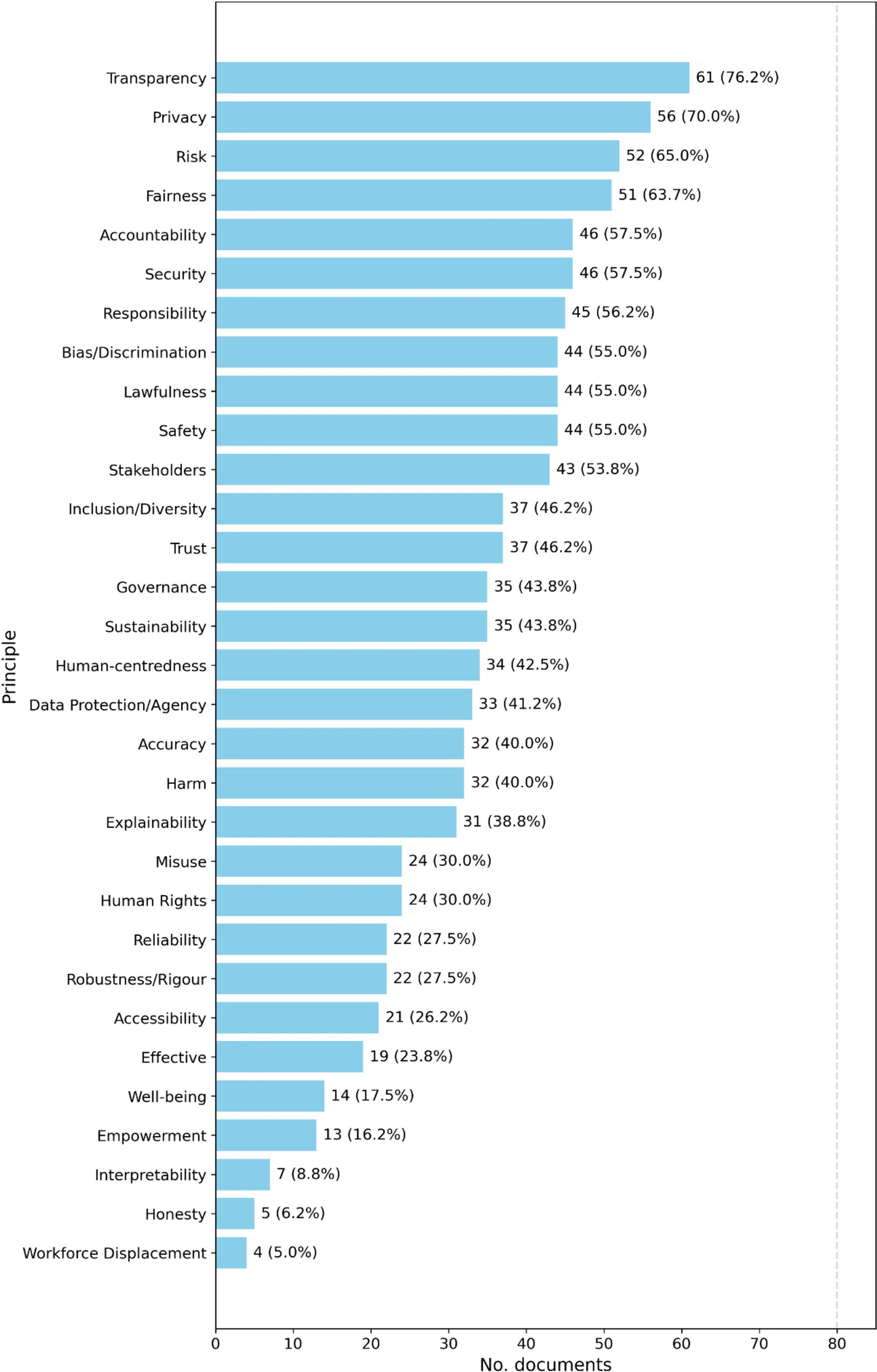
Frequency of principles across all 80 documents analysed. For each principle, the total number of documents is given with the percentage of the corpus covered. The dashed line indicates full coverage in all 80 documents, which we did not observe for any individual principle

We observed high variability in terms of the responsible AI principles addressed in the documents in our collection (Fig. 5). This clearly illustrates the fragmentation of responsible AI across different policies and organisations. The most frequent concept, Transparency, appeared in only 61 of the 80 documents (76%), and Workforce Displacement was only mentioned in 4 (5%). A number of previous studies have treated the principles occurring in more than 50% of the documents as a basis for identifying a consensus around a core set of principles. The eleven terms occurring in more than 50% of our corpus were: “Transparency, Privacy, Risk, Fairness, Accountability, Security, Responsibility, Lawfulness, Bias/discrimination, Safety, and Stakeholders”. Table 3 illustrates the differences between our most frequent findings and those of prior studies.

**Table 3:** Most common principles emphasised for RAI, comparing prior literature and this study

Jobin et al. (2019)	Fjeld et al. (2020)	Hagendorff (2020)	Corrêa et al. (2023)	This study
Transparency	Fairness and non-discrimination	Privacy protection	Transparency/explainability/auditability	Transparency
Justice, fairness and equity	Privacy	Fairness, non-discrimination and justice	reliability/safety/security/trustworthiness	Privacy
Non-maleficence	Accountability	Accountability	Justice/equity/fairness/non-discrimination	Risk
Responsibility and accountability	Transparency and explainability	Transparency, openness	Privacy	Fairness
Privacy	Safety and security	Safety, cybersecurity	Accountability/liability	Accountability
+ 6 other principles that appear in less than 50% of documents (Beneficence, freedom and autonomy, trust, sustainability, dignity, solidarity)	Professional responsibility	Common good, sustainability, well-being	Freedom/autonomy/democratic values/technological sovereignty	Security
	Promotion of human values	Human oversight, control, auditing	+ 11 other principles that appear in less than 50% of documents	Responsibility
	Human control of technology	+ 15 other principles that appear in less than 50% of documents		Lawful
				Bias/discrimination
				Safety
				Stakeholders
				+ 20 other principles that appear in less than 50% of documents

The treatment of 50% as a cut-off point may be simplistic, but it does help suggest that there might be a set of principles that are relevant in many sectors. Examining our list, it is useful to note that several of these terms, such as transparency, accountability, and privacy, have been found to frequently occur in previous studies. These also echo the widely cited FATE principles (Fairness, Accountability, Transparency, and Ethics), which have underpinned much academic effort on responsible AI (Tollon and Vallor 2025). The precise contextual meaning of all these terms may remain partly unclear, but they do constitute key reference points specific to the governance of AI. Thus, the notion of transparency points to the problem of the black-boxing of AI, where how the AI works is not explained to those affected by its use. Accountability points to the problem of determining who is held responsible when things go wrong in decision-making by AI and the risk of deresponsibilisation. Some of the other terms are highly relevant to AI. Bias/discrimination is a frequent issue with AI outputs arising from the unreflective use of biassed historic training datasets. Stakeholders point to the problem of failures of AI development to involve all interested parties in the design of AI. We can see “privacy” and “security” as general concerns around technologies that arise again with AI. “Lawful” implies the risk that new technologies bypass previous legal structures by making assumptions in the law. Again, this may be a common concern about the relation of technologies and the law.

However, certain other terms in the list of 11 most frequent items (and in the wider list) have a very high sense of ambiguity. Terms, such as “risk”, “fairness”, and “responsibility”, have in themselves little specific meaning except in gesturing towards what is bad or good, nor do they have a specific meaning in the context of AI. Of course, we want AI to be fair but the appearance of fairness as a principle only implies that it is a concern that AI might not be, but goes no way to define what fair means in concrete terms. We can speculate that this allows such terms to be used by many actors and to define them in a way that suits the context or indeed their interests. The prevalence of these terms, combined with the first pattern, produces a sense of a consensus of terms without any real conceptual consensus. The problem for practitioners and organisations is how to operationalise them.

Thus, a comparative analysis does suggest some level of cross-sector consensus about some aspects of RAI. However, there is a long tail of infrequently occurring terms that disturbs this apparent consensus. As Hagendorff (2020) establishes, concepts that occur rarely are of considerable interest to gaining an understanding of it. In our data well-being appears in only 17.5% (*n* = 14) of the documents, empowerment in 16% (*n* = 13), interpretability in 9% (*n* = 7), honesty in 6% (*n* = 5), and workforce displacement in 5% (*n* = 4). As regards the latter principle, it is notable that even government statements rarely explicitly address the impact of AI on the labour force. Two other of the terms at the bottom of the list, well-being and empowerment are some of the only positive terms about how AI could have a moral benefit to be found in the list. As other authors have noted, most of the principles for RAI are either about a negative aspect of AI (risk, harm) or a positive attribute of a successful AI system (transparency, privacy), only these two appear to relate positive potential benefits of AI.

### Sector-level variation in principles

**Fig. 6:**
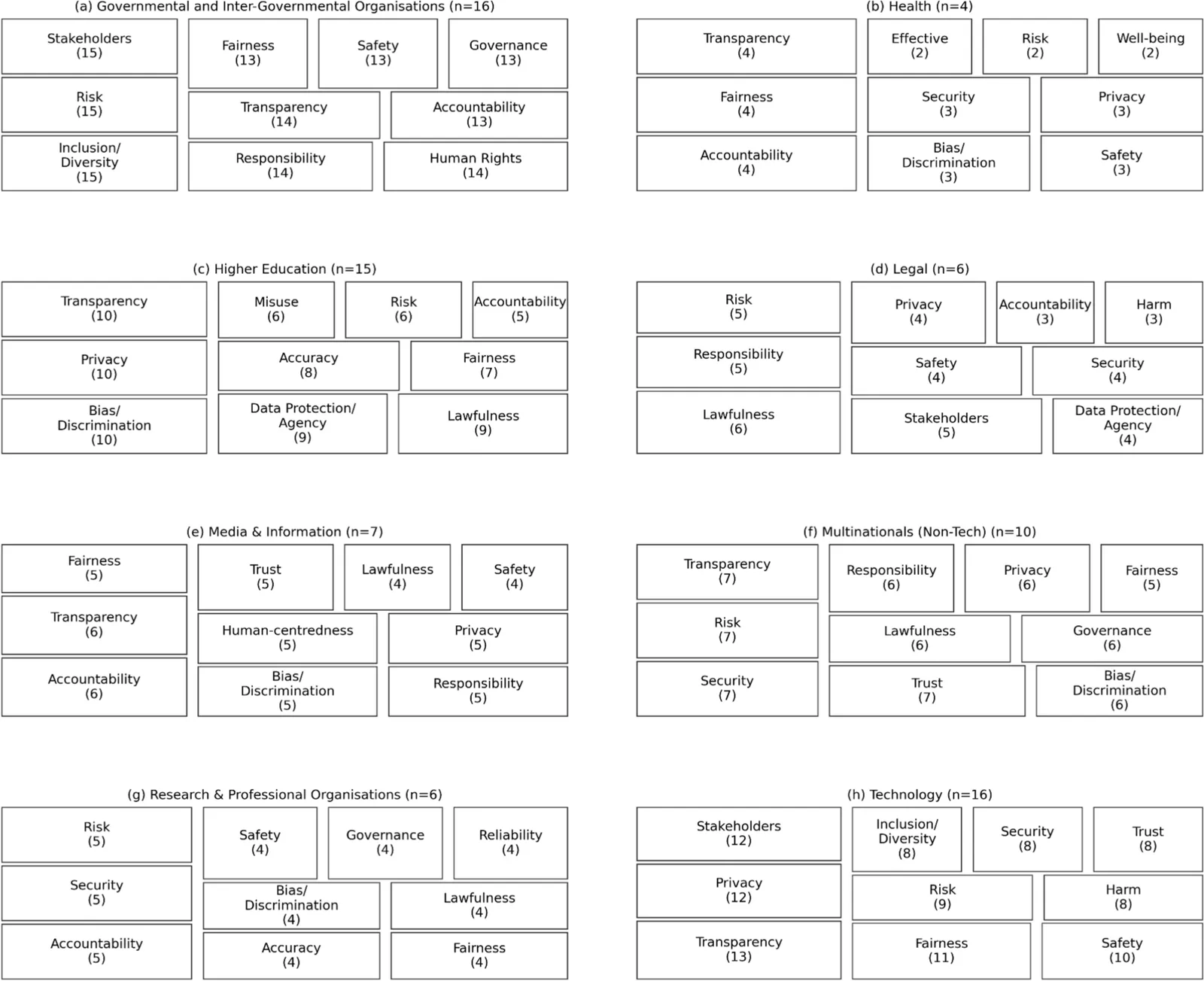
Top-ten most frequent principles by sector, by the number of documents in which each principle appears

Whilst these reflections suggest some commonality across sectors, on the other hand, the most frequent principles per sector, shown in Fig. 6, give a different impression. Although the amount of data in each case is too low to test for statistically significant differences, it does appear that the language being used about AI in the eight sectors being studied is somewhat different. Simply looking at the most frequent concepts, it should not be a surprise that for the Legal sector “lawfulness” is the top item; whilst for Governmental and Inter-governmental Organisations, “inclusion/diversity” and “stakeholders” are the most common principles, together with “risk.” Whilst many of these documents are associated with the European Commission and the United Nations, this remains an unexpected result. There are also certain principles that do seem to have an association with a very particular perspective on responsible AI. For example, the terms inclusion/diversity, human rights, sustainability, and workforce displacement appear to be unambiguously related to the wider societal consequences of AI. Given this association it is not surprising that for the Government sector inclusion/diversity is the most frequently occurring term appearing in all but one of our sample (15/16). Human rights also appears in 14 and sustainability in 13 documents. In contrast, these terms are uncommon in most other sectors, for example, in the Technology sector, suggesting that certain wider social impacts of AI are often forgotten.

Another set of principles seems to be strongly linked to corporate compliance. The most frequent items for the multinational (non-tech) sector are: risk, security, transparency, responsibility, governance, privacy, lawfulness, trust. These terms all resonate with notions of corporate risk management and compliance, though not exclusively. So for this sector it could be suggested that the dominant terms reflect pre-existing sector discourses.

Examining the complete set of frequencies for each principle across the eight sectors (Table 4) highlights marked differences in use for some other terms. The principle of Data Protection/Agency ranges from 12% (Technology) to 60% (Higher Education) and 67% (Legal). Considerations of Safety range from 20% (Higher Education), 30% (Multinationals [Non-tech]) to 81% (Governmental and Inter-governmental Organisations). The principle of Stakeholders ranges from 7% (Higher Education) to 83% (Legal) and 94% (Governmental and Inter-governmental Organisations). The relative concentration of terms differs between sectors as well: only six terms appear in more than 50% of HE documents, compared to nineteen for Governmental and Inter-governmental Organisations.

**Table 4:** Frequency of RAI principles by sector

Principle	Governmental and inter-governmental organisations (*n* = 16)	Health (*n* = 4)	Higher education (*n* = 15)	Legal (*n* = 6)	Media and information (*n* = 7)	Multinationals (non-tech) (*n* = 10)	Research and professional organisations (*n* = 6)	Technology (*n* = 16)	Total
Transparency	14 (88%)	4 (100%)	10 (67%)	3 (50%)	6 (86%)	7 (70%)	4 (67%)	13 (81%)	61
Privacy	12 (75%)	3 (75%)	10 (67%)	4 (67%)	5 (71%)	6 (60%)	4 (67%)	12 (75%)	56
Risk	15 (94%)	2 (50%)	6 (40%)	5 (83%)	3 (43%)	7 (70%)	5 (83%)	9 (56%)	52
Fairness	13 (81%)	4 (100%)	7 (47%)	2 (33%)	5 (71%)	5 (50%)	4 (67%)	11 (69%)	51
Accountability	13 (81%)	4 (100%)	5 (33%)	3 (50%)	6 (86%)	4 (40%)	5 (83%)	6 (38%)	46
Security	13 (81%)	3 (75%)	3 (20%)	4 (67%)	3 (43%)	7 (70%)	5 (83%)	8 (50%)	46
Responsibility	14 (88%)	1 (25%)	4 (27%)	5 (83%)	5 (71%)	6 (60%)	4 (67%)	6 (38%)	45
Bias/Discrimination	8 (50%)	3 (75%)	10 (67%)	3 (50%)	5 (71%)	6 (60%)	4 (67%)	5 (31%)	44
Lawfulness	11 (69%)	1 (25%)	9 (60%)	6 (100%)	4 (57%)	6 (60%)	4 (67%)	3 (19%)	44
Safety	13 (81%)	3 (75%)	3 (20%)	4 (67%)	4 (57%)	3 (30%)	4 (67%)	10 (62%)	44
Stakeholders	15 (94%)	2 (50%)	1 (7%)	5 (83%)	2 (29%)	4 (40%)	2 (33%)	12 (75%)	43
Inclusion/Diversity	15 (94%)	1 (25%)	2 (13%)	1 (17%)	4 (57%)	3 (30%)	3 (50%)	8 (50%)	37
Trust	9 (56%)	2 (50%)	1 (7%)	1 (17%)	5 (71%)	7 (70%)	4 (67%)	8 (50%)	37
Governance	13 (81%)	0 (0%)	1 (7%)	3 (50%)	1 (14%)	6 (60%)	4 (67%)	7 (44%)	35
Sustainability	13 (81%)	1 (25%)	4 (27%)	1 (17%)	3 (43%)	5 (50%)	4 (67%)	4 (25%)	35
Human-centredness	13 (81%)	1 (25%)	0 (0%)	1 (17%)	5 (71%)	4 (40%)	3 (50%)	7 (44%)	34
Data Protection/Agency	9 (56%)	1 (25%)	9 (60%)	4 (67%)	1 (14%)	4 (40%)	3 (50%)	2 (12%)	33
Accuracy	6 (38%)	1 (25%)	8 (53%)	2 (33%)	2 (29%)	4 (40%)	4 (67%)	5 (31%)	32
Harm	12 (75%)	1 (25%)	2 (13%)	3 (50%)	2 (29%)	1 (10%)	3 (50%)	8 (50%)	32
Explainability	11 (69%)	1 (25%)	1 (7%)	3 (50%)	1 (14%)	5 (50%)	3 (50%)	6 (38%)	31
Misuse	6 (38%)	1 (25%)	6 (40%)	3 (50%)	1 (14%)	1 (10%)	2 (33%)	4 (25%)	24
Human Rights	14 (88%)	0 (0%)	1 (7%)	0 (0%)	2 (29%)	0 (0%)	2 (33%)	5 (31%)	24
Reliability	4 (25%)	0 (0%)	5 (33%)	1 (17%)	2 (29%)	2 (20%)	4 (67%)	4 (25%)	22
Robustness/Rigour	7 (44%)	0 (0%)	4 (27%)	2 (33%)	1 (14%)	2 (20%)	2 (33%)	4 (25%)	22
Accessibility	7 (44%)	1 (25%)	3 (20%)	1 (17%)	1 (14%)	2 (20%)	1 (17%)	5 (31%)	21
Effective	4 (25%)	2 (50%)	5 (33%)	1 (17%)	3 (43%)	1 (10%)	3 (50%)	0 (0%)	19
Well-being	7 (44%)	2 (50%)	1 (7%)	0 (0%)	0 (0%)	1 (10%)	2 (33%)	1 (6%)	14
Empowerment	6 (38%)	0 (0%)	1 (7%)	0 (0%)	2 (29%)	1 (10%)	0 (0%)	3 (19%)	13
Interpretability	3 (19%)	0 (0%)	0 (0%)	1 (17%)	0 (0%)	0 (0%)	2 (33%)	1 (6%)	7
Honesty	1 (6%)	0 (0%)	2 (13%)	0 (0%)	0 (0%)	0 (0%)	1 (17%)	1 (6%)	5
Workforce Displacement	0 (0%)	0 (0%)	1 (7%)	0 (0%)	0 (0%)	0 (0%)	2 (33%)	1 (6%)	4

**Fig. 7:**
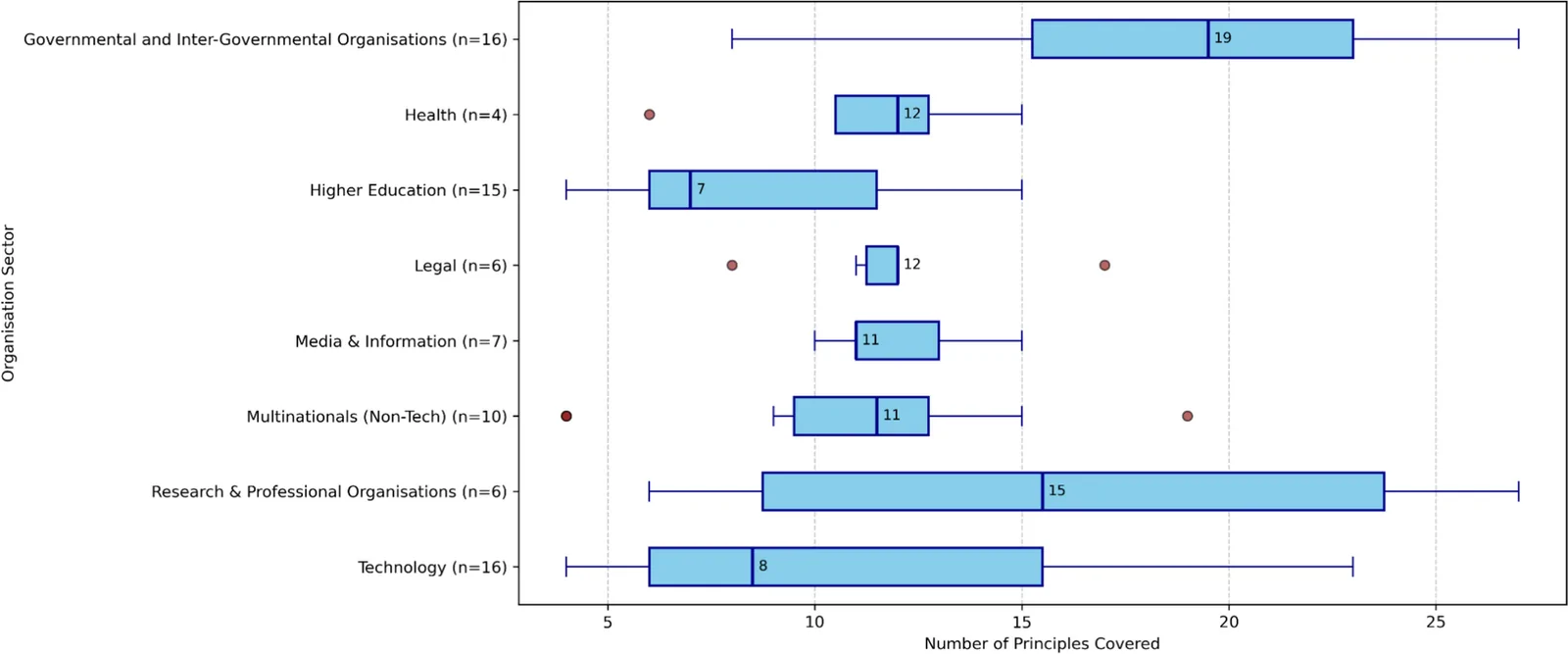
Distribution of the number of principles identified in each document, by sector. The median number of unique principles per document is shown

Figure 7 illustrates this pattern more broadly, with variation between sectors in the number of distinct principles addressed within each document. Whilst some of this is undoubtedly due to variation in document length (e.g. many government policy documents are extensive), we found most of the variation to be in content. Documents in the Technology sector tended to mention a smaller number of RAI principles (median of 9), but with high variation across the 16 documents analysed. Governments and Research & Professional Organisations exhibited similarly high variation, but with a higher number of principles mentioned. By contrast, Health, Legal, Media & Information, and Multinationals (Non-tech) all exhibited much lower variation in the number of principles, with consistent medians of 11 or 12 distinct principles. This shows an intriguing variation not only in the kinds of RAI principles sectors speak to, but the diversity of principles they represent as relevant for their audiences.

### Changes from generative AI

**Fig. 8:**
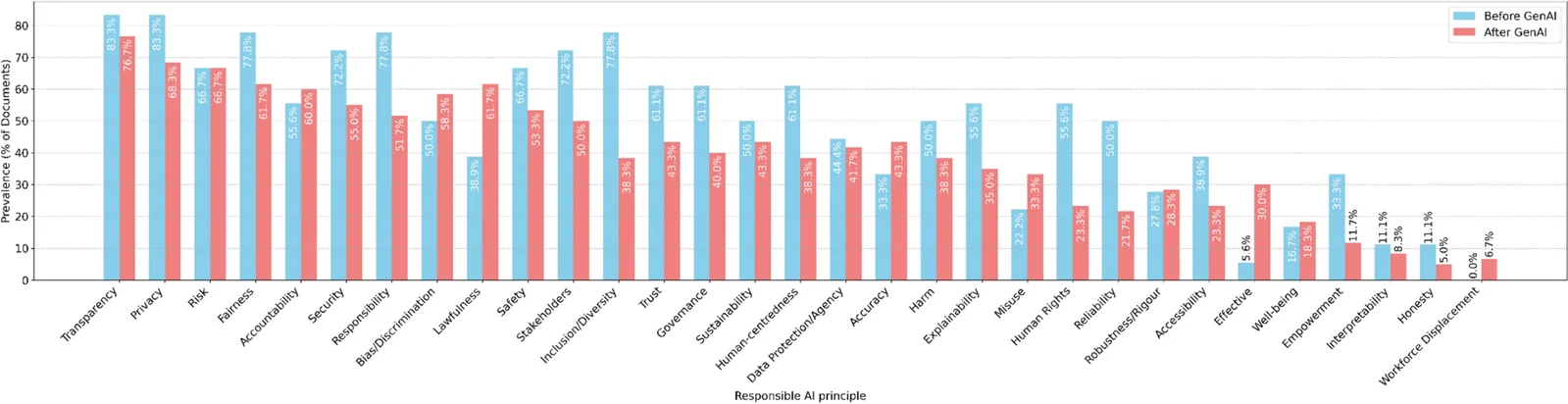
Prevalence of RAI principles before and after commercially available generative AI

To assess the impact of commercially available generative AI technologies on responsible AI principles, we measured the prevalence of each of our 31 principles in documents published before the end of 2022 and 2023 forwards. Whilst ChatGPT was publicly launched in late November 2022, we allow for a 1-month lag time in producing new policies. As shown in Fig. 8, there is a marked shift in language between the 18 pre-2023 documents and the 62 documents published from 2023 onwards. Terms related to practical and legal impacts of AI use, such as lawfulness, misuse, accuracy, and effectiveness, are observed in an increased share of documents post-ChatGPT, suggesting a possible shift in recognition of tangible impacts of AI use. However, other related principles, such as reliability, safety, and even responsibility, decrease in prevalence, indicating this is not a consistent effect. Notably, both observed mentions of ‘workforce displacement’ are in documents published post-ChatGPT.

## Discussion

Our findings clearly illustrate the fragmented nature of responsible AI at a key moment in its recent development. As AI use has continued to proliferate and new AI models and providers have entered the market, this fragmentation has almost certainly grown and perhaps even accelerated. Our analysis shows the importance of getting more specific about what responsible AI should mean in practice, to move from broad statements to more tangible shared reference points.

Moreover, we have clearly demonstrated the level of variation observed in AI policies at a sector level, illustrating the need for future policy development to be sensitive to the specific needs and characteristics of different sectors. Whilst our findings show fragmentation, not all of that fragmentation is harmful: as we have observed, different sectors address different stakeholders and contexts for AI use, and this sensitivity to context is also an essential part of addressing responsible AI. This paper provides clear evidence for the need to account for sector context in responsible AI, and the need for further research to better understand how responsible AI varies in practice amongst different sectors.

Previous work has tended to perceive a trend towards consensus around certain core principles for RAI. In contrast, we draw attention to fragmentation and continuing multiplicity and divergence. On the one hand, the long-standing and society-wide debate does create salience to particular terms that do have many mentions (without necessarily being interpreted in the same way). But our data shows that in different sectors different sets of principles are seen as important. Sectors themselves have some commonality but there are marked differences between them. In addition, changes in the technology shift perceptions of risk and so the principles at stake point to temporal change as occurring on top of sector difference. Our study draws attention to the need to acknowledge and study sector and temporal variation and not just focus on frequently mentioned terms at a global level. It recognises that RAI remains a far from clear concept and points to the importance of examining sectoral/local conditions for understanding how AI is operationalised. It supports the idea that policy should be sensitive to sectoral needs. Some of the differences are immediately understandable. Governmental and Inter-governmental Organisations stress society-wide concerns, far less referenced by most other sectors. The legal sector gives greater stress to legal compliance. Further research is needed to understand how this variation is generated as a process and its implications for practice.

### Learning from a specific moment in responsible AI

In contrast to previous studies, our analysis provides focussed learnings about the language attached to RAI in a specific geopolitical context, namely the UK. Our document collection does include statements made by organisations based in other countries, for example those by the Big U.S. Tech companies that dominate the UK technology environment, reflecting the inherently international nature of the current AI ecosystem. The relevance of our findings is thus not restricted to the UK alone, but reflects the broader exchange between international and multinational actors and local or national organisations in shaping responsible AI in practice. Notably, our study is based purely on organisational statements: it does not include academic literature, unlike Hagendorff (2024). Our findings are thus more closely rooted in organisational policy and practice, reflecting the challenges of responsible AI ‘on the ground.’

Our sample is a snapshot of principles that were current in the UK in the mid-2024 though some had been written a considerable time before that. We do observe a new flurry of AI ethics statements during 2023, reflecting generative AI’s impact on how AI was perceived. For example, nearly all the HE and healthcare statements were dated from this period. The previous high point was 2018 (Corrêa et al. 2023). This seems to confirm Hagendorff’s (2024) claim that interest in RAI had shifted with generative AI. The frequency of publication of responsible AI documents and the responsiveness of many documents we analysed to the specific challenges of generative AI illustrate the nature of responsible AI as a moving target. By analysing a moment of significant change in AI, our study provides more insight into how responsible AI will continue to change, and the need to consider diverse contexts and stakeholders engaged with shaping responsible AI in practice.

### Understanding the complexity of responsible AI

A wide range of principles were in use for RAI: 31 different principles were identified in this study. Transparency was the commonest principle (76% of documents), confirming previous studies where that term (and related concepts) was similarly the most-cited principle. However, our other most common principles differ somewhat from prior literature. Comparing to Corrêa et al.'s (2023) listing as the most recent similar study (but pre generative AI), our findings are largely similar in terms of the identification of commonly mentioned principles (see Table 3), but lack reference to terms, such as freedom and autonomy, and include the issue of stakeholder engagement. This suggests that although generative AI shifted views of RAI, it has had limited impact on the underlying principles of RAI as shown by the relatively low degree of shift in the language used. This does suggest a sense of continuity, albeit, as several previous authors comment, the contextual meaning of these terms may vary. Since several of these terms like “responsible” or “fairness” are rather vague or require contextual clarification, part of the challenge of defining RAI appears to lie in the use of rather abstract terms.

The proliferation of additional terms beyond this core is also likely to be part of the challenge of how any individual organisation can get a clear fix on RAI when context is so crucial. We identified 20 other terms that appear in less than 50% of all the documents. Corrêa et al. (2023) who had 17 different clusters of principles in total, had 11 appearing in less than 50% of documents. Far less frequently mentioned terms in our data include, first, terms relating to global governance such as “human rights”. Second, terms relating to social and global harms, such as “workforce displacement.” It remains surprising that impacts on employment seem to be so little emphasised in RAI documents. It should be noted, however, that “sustainability” is mentioned in 35 documents, and “inclusion and diversity” is in 37, so not far short of 50% of the documents. Third, lacking are terms relating to individual benefit, such as “well-being” and “empowerment”. Perhaps these do not get frequent mention because they are positive aspects, and most terms are about the risks of AI. As other authors note the statements of principle dwell on the negative dimension of AI not the ethical benefits of uses (Jobin et al. 2019; Hagendorff 2020). Also, as Hagendorff (2020) points out, there remain societal concerns that are not mentioned at all in any policy statements, e.g. the impact of AI on social cohesion or political misuse of AI.

### Sector matters: the context sensitivity of responsible AI

One of the current study’s main aims was to interrogate sectoral differences. The presence of differences by sector is confirmed in terms of some notable divergences in terms of audience, timing and content. The statements address different types of audiences, with most HE documents focussed on the organisation itself, many others explicitly addressing a sector. The timing of publication seemed to vary by sector too. HE and health seemed to be triggered by generative AI, whereas the technology company statements were more spread over time. Critically, there were also differences by sector in the most frequently mentioned principles. Certain terms, such as inclusion/diversity and human rights, sustainability appeared very commonly in government documents, and much less frequently in technology company or HE statements. We also argue that in the multinational (non-tech) sector there is a focus on terms, such as security, governance, privacy, and lawfulness in alignment with the typical language of corporate risk management.

Our sectoral analysis illustrates the difficulty of making sense of RAI given the multiplicity of terms in use, the variation in sectoral language and the ambiguity of many of the terms. Because our study actively sought to generate a collection of documents to examine some important UK sectors rather than use a pre-existing collection, it is possible to identify that some sectors were not producing any publicly available statements. For example, they were less frequent amongst news media organisations than might be expected. This could be because the sector only tends to publish principles internally, but might also indicate less attempt to define principles. Other sectors tended to be reliant on sector-level statements, e.g., Law.

### Limitations

We followed conventional methods in searching for items (principles) to analyse, but there will always be variations in success that mean at a different time a searcher might have found or not found other items. There is not a way to be completely systematic or gather a comprehensive collection of statements of principles when these are not formally published. There are also differing sharing conventions, so we cannot be sure that *not* finding items is the result of items not being openly published rather than not existing. Based on our contextual knowledge, we selected the sample organisations based on size, reputation, and influence. Whilst this might be deemed to include an element of subjectivity, we provide a complete list of the organisations selected in the appendix. The relatively low total numbers of documents precludes rigorous statistical measurement of differences, particularly between sectors. Nevertheless, we believe our approach to collecting items does enhance our understanding and may be usefully emulated in the future studies.

## Conclusion

The rapid development of AI and its related concerns call for social action to ensure responsible AI. Understanding responsible AI is a key prerequisite to putting it into practice. Our analysis of 80 documents setting forth organisational perspectives, policies, and statements on AI clearly demonstrates that current understanding of responsible AI is fragmented, changing and highly variable. We have shown that sector differences are a key component of variation in responsible AI, establishing the importance of accounting for sector context in the future research and policy development.

Our study adds to the responsible AI literature by providing much-needed context and a deeper understanding of the role of sector, place, and time in shaping how responsible AI is realised in policy documents. Many organisations have sought to articulate principles for RAI in the last five years, reflecting both a belief in AI’s potential benefit and profound concerns about its ethical and other implications. In response, there have been several academic attempts to review, even synthesise these listings of principles. Unlike previous analyses, we have explicitly focussed on examining sector differences, and specifically examined the UK context. The 80 documents from eight sectors relevant to the UK include 31 different principles, of which eleven appear in more than 50% of the publications. Some of these 11 align to those found in previous studies. However, there are also 20 principles that are less frequently mentioned, and some reference points that are never mentioned. There do appear to be marked differences of emphasis by sector, in terms used and emphasis, as well as intended audience and timing. Governmental statements of principle have a very different flavour from multinationals or education. This finding reveals the need for more recognition that RAI must be discussed at the sector level. One reason for the uncertainty amongst AI developers about ethical practice is diverging lists of principles, as well as the ambiguity of some of the terms in use. We suggest that greater clarity for practice can be achieved when sector differences are acknowledged. It is also noted that there did seem to have been a change caused by the advent of generative AI. Some sectors were prompted to produce policies and some principles seemed to gain more emphasis after this.

This study reflects the UK at a particular point in time. Since the time the data were collected, a new government has been elected both in the UK (and in the USA) and there have been potentially significant shifts in AI technologies, such as the launch of a Chinese competitor in the generative AI space, Deepseek. The agenda around the sustainability of AI seems to have gained more prominence in the media. These changes could potentially have already shifted feelings about key AI principles. However, we do not think our findings about sector variance are likely to be irrelevant in other temporal or geographical contexts. Our study demonstrates an important pattern in thinking about RAI, but we need more dynamic ways to capture the development of RAI. Notwithstanding this, we share the concern of Roche et al. (2023) and others that Global South voices are under-represented in discussion of AI, and our current study does not address this limitation. Future research could usefully draw out comparisons with RAI in other contexts, such as Latin America or Africa.

Our study demonstrates that rather than coalescing around consensus, responsible AI is a fundamentally fragmented landscape. Importantly, these differences may in fact be useful in putting responsible AI into practice. Differences between organisations and sectors reflect, amongst other things, differences in what is perceived to be actionable in responsible AI for people and organisations in that sector, and variations in the contexts in which people work. Future discussion of responsible AI, and efforts to develop RAI practice, must be sensitive to these differences and the need for approaches that are sensitive to different sectoral and organisational contexts.

## Supplementary Information

Below is the link to the electronic supplementary material.

Supplementary file1 (DOCX 23 KB)

## Data Availability

No datasets were generated or analysed during the current study.
