# Supplementary material for: The fragmentation of responsible AI: sector variation in organisational AI policies and statements of principle
Source: AI Soc. 2026 Jun 27;41(10):9899–917. doi: 10.1007/s00146-026-03175-7 (PMC13690845; doi:10.1007/s00146-026-03175-7)
Supplement: Supplementary file 1 [file 146_2026_3175_MOESM1_ESM.docx]

**Appendix**

**Appendix: Full list of organisational AI policies analysed**

| **Document title** | **Publisher Name** | **Organisation Sector** | **Publication Date** |
| --- | --- | --- | --- |
| Seoul Declaration for safe, innovative and inclusive AI by participants attending the Leaders' Session: AI Seoul Summit, 21 May 2024 | AI Seoul Summit | Governmental and Inter-Governmental Organisations | May 2024 |
| AI Foundation Models: Update paper | Competition & Markets Authority | Governmental and Inter-Governmental Organisations | April 2024 |
| AI Act (classifies AI systems according to their risk) | EC | Governmental and Inter-Governmental Organisations | February 2024 |
| THE ASSESSMENT LIST FOR  TRUSTWORTHY ARTIFICIAL  INTELLIGENCE (ALTAI)  for self assessment | EC | Governmental and Inter-Governmental Organisations | July 2020 |
| Ethics guidelines for trustworthy AI | EC | Governmental and Inter-Governmental Organisations | April 2019 |
| Responsible AI Working Group Report | Global Partnership on Artificial Intelligence | Governmental and Inter-Governmental Organisations | December 2023 |
| Artificial intelligence | ICO | Governmental and Inter-Governmental Organisations | March 2023 |
| OECD AI Principles Overview | OECD | Governmental and Inter-Governmental Organisations | May 2024 |
| Recommendation of the Council on Artificial Intelligence | OECD | Governmental and Inter-Governmental Organisations | May 2024 |
| A pro-innovation approach to AI regulation: government response | UK government | Governmental and Inter-Governmental Organisations | February 2024 |
| Interim report: Governing AI for Humanity | UNESCO | Governmental and Inter-Governmental Organisations | December 2023 |
| Ethics of Artificial Intelligence | UNESCO | Governmental and Inter-Governmental Organisations | May 2022 |
| Recommendation on the Ethics of Artificial Intelligence | UNESCO | Governmental and Inter-Governmental Organisations | November 2021 |
| Seizing the opportunities of safe, secure and trustworthy artificial intelligence systems for sustainable development | United Nations | Governmental and Inter-Governmental Organisations | March 2024 |
| Principles for the ethical use of artificial intelligence in the United Nations system | United Nations | Governmental and Inter-Governmental Organisations | October 2022 |
| Principles for the Future of Responsible Media in the Era of AI | World Economic Forum | Governmental and Inter-Governmental Organisations | January 2024 |
| Privacy notice | Bupa | Health | November 2023 |
| Artificial Intelligence in Health, Health Care, and Biomedical Science: An AI Code of Conduct Principles and Commitments Discussion Draft | National Academy of Medicine | Health | April 2024 |
| Artificial Intelligence | NHS | Health | February 2023 |
| WHO calls for safe and ethical AI for health | World Health Organisation | Health | May 2023 |
| Using generative AI (DMU library) | De Montfort University | Higher Education | May 2024 |
| GCU guidance on appropriate use of generative AI by students for Education purposes, November 2023 | Glasgow Caledonian University | Higher Education | November 2023 |
| Principles for the use of AI in FE colleges | Jisc | Higher Education | April 2024 |
| Leeds Beckett's guiding principles for the use of generative artificial intelligence(AI) | Leeds Beckett University | Higher Education | [Unknown month] 2024 |
| New principles on use of AI in education | Russell Group | Higher Education | July 2023 |
| AI and you: learning and assessment | Sheffield Hallam University | Higher Education | May 2024 |
| Student guidance on using Generative Artificial Intelligence tools ethically for study | University of Birmingham | Higher Education | July 2023 |
| General Principles for Use of Generative AI | University of Cambridge | Higher Education | February 2024 |
| Guidance on the Use of Artificial Intelligence (AI) | University of Greenwich | Higher Education | June 2023 |
| Policy on Generative Artificial Intelligence in Learning,  Teaching and Assessment | University of Leicester | Higher Education | May 2024 |
| Artificial Intelligence (AI) Teaching Guidance | University of Manchester | Higher Education | October 2023 |
| Use of generative AI tools to support learning | University of Oxford | Higher Education | January 2024 |
| Principles for Generative AI in learning and teaching | University of Sheffield | Higher Education | April 2024 |
| POLICY IN RELATION TO THE SAFE USE OF GENERATIVE AI (GenAI) | University of Westminster | Higher Education | July 2023 |
| Use of generative Artificial Intelligence in PGR programmes | University of York | Higher Education | June 2023 |
| The AI Series | Ashurst | Legal | September 2023 |
| Artificial Intelligence (AI) is transforming legal services and the wider services industry. | Clifford Chance | Legal | [Unknown month] 2024 |
| Generative AI: Five things for lawyers to consider | Freshfields Bruckhaus Deringer | Legal | February 2023 |
| Linklaters AI Charter | Linklaters | Legal | October 2023 |
| Guidance for Implementing Responsible AI in Legal and Business Practice | Ropes & Gray | Legal | May 2024 |
| Regulating AI | Slaughter & May | Legal | June 2023 |
| Research Libraries Guiding Principles for Artificial Intelligence | Association of Research Libraries | Media & Information | April 2024 |
| ARL/CNI AI Scenarios: AI-Influenced Futures | Association of Research Libraries/Coalition for Networked Information | Media & Information | June 2024 |
| BBC AI Principles | BBC | Media & Information | February 2024 |
| Generative AI at the BBC | BBC | Media & Information | October 2023 |
| Responsible AI Principles | Elsevier | Media & Information | December 2023 |
| Global Principles on Artificial Intelligence (AI) | News Media Association | Media & Information | August 2023 |
| Data and AI ethics principles | Reuters | Media & Information | November 2022 |
| Accenture’s blueprint for responsible AI | Accenture | Multinationals (Non-Tech) | May 2024 |
| AI Ethics & Governance | Accenture | Multinationals (Non-Tech) | May 2021 |
| Responsible AI | Boston Consulting Group | Multinationals (Non-Tech) | September 2023 |
| Proactive risk management in Generative AI | Deloitte | Multinationals (Non-Tech) | June 2023 |
| Trustworthy AI | Deloitte | Multinationals (Non-Tech) | April 2020 |
| EY’s commitment to developing and using AI ethically and responsibly | EY | Multinationals (Non-Tech) | September 2023 |
| Going all in with AI? How to keep the customer at the center | Ipsos | Multinationals (Non-Tech) | November 2023 |
| Responsible AI: Trusted use of data and artificial intelligence | KPMG | Multinationals (Non-Tech) | June 2024 |
| Responsible AI at PwC: Building and operating AI you can trust | PWC | Multinationals (Non-Tech) | June 2019 |
| The Aletheia Framework®: Helping build trust in artificial intelligence | Rolls Royce | Multinationals (Non-Tech) | December 2021 |
| Ada Lovelace Foundation models  in the public sector | Ada Lovelace Institute | Research & Professional Organisations | May 2023 |
| Explainer: A Sociotechnical Approach to AI Policy | Data & Society | Research & Professional Organisations | May 2024 |
| IEEE Ethically aligned design | IEEE | Research & Professional Organisations | March 2019 |
| ISO/IEC 42001:2023 | ISO | Research & Professional Organisations | December, 2023 |
| Understanding artificial intelligence ethics and safety | The Alan Turing Institute | Research & Professional Organisations | June 2019 |
| Science in the Age of AI | The Royal Society | Research & Professional Organisations | May 2024 |
| Transform responsible AI from theory into practice | Amazon Web Services | Technology | September 2023 |
| The new Bing: Our Approach to Responsible AI | Bing | Technology | February 2023 |
| Cisco Principles for Responsible Artificial Intelligence | CISCO | Technology | January 2022 |
| The Cisco Responsible AI Framework | CISCO | Technology | January 2022 |
| Google AI Principles | Google | Technology | January 2024 |
| Responsible AI practices | Google | Technology | May 2023 |
| AI Ethics | IBM | Technology | July 2023 |
| Responsible AI | Intel | Technology | September 2022 |
| Responsible AI: Driven by our belief that AI should benefit everyone | Meta | Technology | July 2023 |
| Meta Llama: Responsible Use Guide | Meta | Technology | April 2023 |
| Microsoft Responsible AI Standard, v2 | Microsoft | Technology | June 2022 |
| Microsoft guidelines for Human-AI interaction | Microsoft | Technology | June 2019 |
| Product safety standards | OpenAI | Technology | February 2023 |
| Generative AI: 5 Guidelines for Responsible Development | Salesforce | Technology | February 2023 |
| Trusted AI | Salesforce | Technology | January 2022 |
| SAP responsible AI handbook | SAP | Technology | August 2023 |
